# Rapidly progressive cognitive impairment: an unusual presentation of cerebral venous thrombosis caused by JAK2 V617F-positive primary myelofibrosis: A case report

**DOI:** 10.1097/MD.0000000000021757

**Published:** 2020-08-21

**Authors:** Chongyao Jin, Jiali Pu, Zhijian Zhou, Xia Chen, Jimin Wu, Baorong Zhang

**Affiliations:** a Department of Neurology, Second Affiliated Hospital, College of Medicine, Zhejiang University, Hangzhou; b Department of Neurology, Affiliated Shaoxing Hospital of Traditional Chinese Medicine, Zhejiang Chinese Medical University, Shaoxing, Zhejiang; c Department of Neurology, Shangrao People's Hospital, Jiangxi, China.

**Keywords:** case report, cerebral venous thrombosis, cognitive impairment, JAK2 V617F, primary myelofibrosis

## Abstract

**Rationale:**

Cerebral venous thrombosis (CVT) is a rare cerebrovascular condition, which mainly manifests as headaches, seizures, and focal neurological deficits. JAK2 mutation in myeloproliferative diseases increases the risk of CVT.

**Patient concerns:**

This 40-year-old woman suffered from rapidly progressive cognitive impairment and limb weakness. Her symptoms worsened while being treated with mannitol with the diagnose of cerebral hemorrhage.

**Diagnosis:**

The patient was diagnosed with CVT and multiple intracranial hemorrhage caused by JAK2 V617F mutation-positive primary myelofibrosis by neuroimage and whole-exome sequencing.

**Intervention:**

She received low-molecular-weight heparin sodium 3800 IU twice a day followed by oral anticoagulant therapy.

**Outcomes:**

The patient showed full recovery from limb weakness and in the follow-up period she noticed no change in her memory.

**Lessons:**

Clinicians should be aware of the possibility of the JAK2 V617F mutation in CVT patients without known causes or risk factors

## 1 Introduction

Cerebral venous thrombosis (CVT) is a major cause of stroke and affects about 1 to 3 people per million.^[1]^ The key clinical manifestations of CVT are headaches, seizures, and focal neurological deficits.^[2]^ Rapidly progressive cognitive impairment is rare as the first symptom of CVT. The etiology of CVT is multifactorial and includes acquired and genetic factors. The JAK2 V617F (c.1849G>T, p.Val617Phe) mutation is known to be involved in the development of CVT.^[3]^ It may influence coagulation by increasing the number of blood cells and altering the functions of platelets and some coagulation factors.^[4,5]^ In addition, the JAK2 V617F mutation^[6,7]^ is common in Philadelphia-negative myeloproliferative neoplasms (MPNs), including polycythemia vera, essential thrombocythemia, and primary myelofibrosis (PMF).^[6]^ Patients with MPNs show a higher morbidity rate for CVT, while compared with essential thrombocythemia and polycythemia vera, CVT due to JAK2 V617F-positive PMF is rare.^[7–10]^

Here, we describe an unusual case of a 40-year-old woman with rapidly progressive cognitive impairment and limb weakness, diagnosed with CVT accompanied by JAK2 V617F-positive PMF. The patient and her family explicitly agreed to her inclusion in this investigation and provided written informed consent for publication.

## 2 Case report

A 40-year-old woman was admitted to our hospital for rapidly progressive cognitive decline for 15 days and left limb weakness for 5 days. She was diagnosed with cerebral hemorrhage in the local hospital and treated with mannitol (125 mL q8 h). However, her symptoms aggravated and she became drowsy and refused to eat.

On physical examination, she showed lethargy, disorientation and memory impairment, left limb weakness, and positive Babinski sign. Enlargement of the spleen and liver was detected on abdominal palpation.

**Figure 1:**
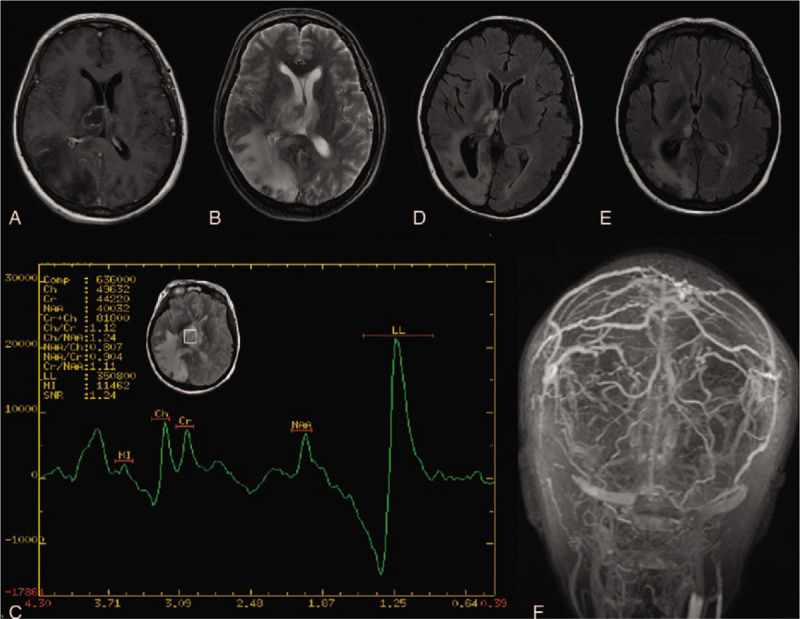
Neuroimaging examination of the patient. (A) MRI T1 and (B) MRI T2 indicated right hemorrhage and edema thalamus and occipital lobe venous F, MRV of sagittal and coronal section showed thrombosis of parietooccipital segment of superior sagittal sinus, right transverse sinus, sigmoid sinus. (C) MRS showed an increased lipoprotein level and a normal cholesterol level in the right thalamus lesion, indicating necrosis. (D and E) MRI T2 Flair of a forth months and ninth months follow-up MRI showed a decreased lesion in the right thalamus and occipital lobe. MRI = magnetic resonance imaging, MRS = magnetic resonance spectroscopy, MRV: magnetic resonance venography.

Laboratory testing showed a leukocyte count of 20.7 × 10^9^/L (normal level, 4.0–10.0 × 10^9^/L) and a platelet count of 531 × 10^9^/L (normal level, 100–300 × 10^6^/L). Tumor marker testing showed an elevated neuron-specific enolase level of 47.8 ng/mL (normal level, <25 ng/mL). Antineutrophil cytoplasmic antibody, antinuclear antibody, and other autoimmune antibody levels were normal. Prothrombin time level was 15.6 seconds (normal level, 12.0–14.0 seconds), and activated partial thromboplastin time was 59.2 seconds (normal level, 30–45 seconds) Lumbar puncture was performed and intracranial pressure was found to be 200 mmH_2_O; the protein level in the cerebrospinal fluid was 104.10 mg/dL (normal level, 8–43 mg/dL). Brain magnetic resonance imaging (Fig. 1A and B) indicated right thalamic and occipital hemorrhage and edema. Magnetic resonance venography (Fig. 1F) showed thrombosis of the parieto-occipital segment of the superior sagittal sinus, right transverse sinus, sigmoid sinus, internal jugular vein, and left transverse sinus vein. Therefore, she was diagnosed with CVT and multiple intracranial hemorrhage. Furthermore, an abdominal computed tomography with contrast was performed and it showed a giant spleen, portal hypertension, a small amount of hydrocele fluid in the abdomen and pelvis. In addition, magnetic resonance spectroscopy focusing on the right thalamic (Fig. 1C) lesion reported an increased lipoprotein level and a normal cholesterol level, indicating necrosis instead of tumor development.

Because the patients had multiple systemic conditions, she underwent whole-exome sequencing, which showed a mutation in JAK2 V617F. Bone marrow puncture was subsequently performed, and the cytological examination revealed myeloid hyperplasia with increased neutrophils alkaline phosphatase rate while bone marrow biopsy showed hematopoietic hyperplasia and reticular fiber hyperplasia.

The patient was eventually diagnosed as showing CVT accompanying multiple intracranial hemorrhage with JAK2 V617F positive PMF. She received treatment with low-molecular-weight heparin sodium 3800 IU twice a day. One week later, she showed a significant improvement in symptoms and was discharged with continued oral anticoagulant therapy. Follow-up brain magnetic resonance imaging scans at 4 (Fig. 1D) and 9 months (Fig. 1E) showed a reduction signal in the right thalamic and occipital lobe lesion. The patient showed full recovery from limb weakness. As for her cognitive, the patient herself said she did not feel any change in her memory and was able to continue her work as usual, and the family number did not notice any change in her personality or behaviors.

## 3 Discussion

We report a rare case of rapid progressive cognitive decline and limb weakness diagnosed with CVT and multiple intracranial hemorrhage caused by JAK2 V617F mutation-positive PMF, which led to systemic hypercoagulability. In the literature review, the accurate prevalence of JAK V617F positive PMFs developing into CVT is currently unknown.

The mechanism of vascular thrombosis related to JAK2 V617F mutation is not well understood. In the classical theory, the pathogenesis of thrombosis is described by 3 main factors: abnormalities of the vessel wall, blood components, and the dynamics of flow. In addition, JAK2 V617F influences the function of JAK2 by constitutive activating this receptor and its downstream effectors, leading to stimulation of cell proliferation, differentiation, and migration.^[11]^ This process can lead to increased levels of erythrocytes and leukocytes, which are potential risk factors for thrombosis.^[12]^ Besides an increase in cell number, platelet function is also altered. One study using a JAK2V617F knock-in mouse model showed changes in both platelet and megakaryocyte activities. Megakaryocytes became more hypersensitive to endogenous stimulants such as fibrinogen and thrombopoietin. Platelets, meanwhile, exhibit increased thrombus formation as well as enhanced reactivity to thrombin and aggregation,^[4]^ leading a more thrombosis-vulnerable status. In addition, leucocytes may also play a role in embolism in JAK V617F mutation patients. Neutrophils expressing JAK2-V617F show increased activation of β1 and β2 integrins, essential mediators of leukocyte adhesion to the endothelium, thereby enhancing thrombus formation.^[13]^ JAK-2 activation is found to up-regulate heparanase, a protein that forms a complex and enhances the activity of tissue factor, which leads to over-production of Xa and subsequent activation of the coagulation system.^[5]^ The JAK2 V617F mutation is also considered to influence the endothelial cells. One study showed that JAK2 V617F endothelial cells display pro-adherent and pro-thrombotic features, which can contribute to the thrombotic events seen in MPN patients.^[14]^

The most common manifestation of CVT is a headache, yet in this case, the patient's first symptom was an impairment of the cognitive function and change in mental status. The reason behind this may be the hemorrhage and edema in the right thalamus, a nuclei closely related to cognition,^[15]^ due to thrombosis of the deep venous system.

Anticoagulation therapy is recommended for venous thromboembolism in patients with MPNs as well as in the general population. Although our patient recovered well from her headache, limb weakness and cognitive impairment and a follow-up magnetic resonance venography examination indicated recanalization of the cerebral vein to some extent. However, as the risk of recurrent venous thromboembolism is high in cases of MPN, relatively prolonged use of anticoagulation may be beneficial.^[16]^ The management of PMF is usually palliative. Observation is recommended for “low-risk” patients, while allogeneic stem cell transplant is preferred for “high-risk” patients. For those “intermediate risk” patients who require treatment, JAK2 inhibitor and some other clinical trial may be considered.^[17]^

In conclusion, rapid cognitive decline as the first symptom of CVT is relatively rare. CVT should be considered in the differential diagnosis when assessing such patients. There are many reasons contributing to CVT and the JAK2 V617F mutation is one of the potential risk factors that acquires the attention of clinicians. Further research on the epidemiology and pathophysiology of JAK2 V617F positive CVT needs to be conducted.

## Author contributions

CJ, ZZ, and XC collected clinical data and drafted the manuscript. JP and BZ contributed to the clinical data collection and clinical management of the patients, and to the manuscript revision. CJ and JW created Figure 1, and contributed to the revision. All authors approved the final version of the manuscript.
